# Substance‐related intrusive memories in cocaine use disorder are different from but associated with craving

**DOI:** 10.1111/add.70449

**Published:** 2026-04-27

**Authors:** Amelie Zacher, Lina Dietiker, Victoria Häffner, Francesco Bavato, Birgit Kleim, Boris B. Quednow

**Affiliations:** ^1^ Experimental Pharmacopsychology and Psychological Addiction Research, Department of Adult Psychiatry and Psychotherapy University Hospital of Psychiatry Zurich, University of Zurich Zurich Switzerland; ^2^ Experimental Psychopathology and Psychotherapy, Department of Psychology University of Zurich Zurich Switzerland; ^3^ Department of Adult Psychiatry and Psychotherapy University Hospital of Psychiatry Zurich, University of Zurich Zurich Switzerland; ^4^ Center of Integrative Functional Neuroscience, Department of Medicine Aarhus University Aarhus Denmark; ^5^ Neuroscience Centre Zurich Joint Centre of University of Zurich and Swiss Federal Institute of Technology Zurich Zurich Switzerland

**Keywords:** addiction memory, cocaine, cocaine use disorder, craving, drug memory, ecological momentary assessment, intrusions

## Abstract

**Background and aims:**

Cocaine use disorder (CUD) is a significant global health concern, characterized by persistent craving despite severe consequences. Recent theories highlight maladaptive memory processes – such as intrusive, vivid recollections of past substance use that arise spontaneously in daily life – as key contributors to craving and relapse; however, empirical studies examining such substance‐related intrusions in naturalistic contexts remain scarce. This study therefore used ecological momentary assessment (EMA) to investigate the frequency, phenomenology and emotional and behavioural correlates of substance‐related memory intrusions in individuals with CUD, and how these relate to craving, cocaine use severity (CUS) and psychotherapy experience.

**Design:**

A 14‐day EMA study captured event‐based reports of intrusions, craving and related experiences in participants diagnosed with CUD.

**Setting:**

The study was conducted in Switzerland in a naturalistic setting, with data collected via individuals' smartphones.

**Participants:**

*N* = 43 participants (recruited in Switzerland, 16% female; 18–59 years old, median compliance rate 82.8%) with a current DSM‐5 diagnosis of CUD made a total of *n* = 360 event‐based reports that were analysed.

**Measurements:**

EMA reports included intrusion episode types (pure intrusions, intrusions with subsequent or simultaneous craving or pure craving), intrusion characteristics (vividness, intrusiveness, modalities), craving intensity, episode triggers and cognitive‐behavioural, emotional and physiological responses. CUS was assessed based on use quantity, duration and obsessive‐compulsive use symptoms.

**Findings:**

Participants recorded an average of 8.4 episodes (standard deviation = 5.8) across 14 days. Intrusions frequently occurred independently of craving (42.4%) but were statistically significantly associated with greater craving intensity when more vivid (unstandardized regression coefficient *b* = 0.53, *P* = 0.002) and intrusive (*b* = 0.48, *P* < 0.001). Episodes involving craving were characterized by greater distress (*b =* 1.52–2.17, all *P* < 0.001) and greater loss of control (*b* = 2.41–3.22, all *P* < 0.001) and were associated with higher odds of reporting obtaining cocaine (odds ratio = 19.90, *P* < 0.001). Higher CUS predicted more frequent intrusion episodes (unstandardized regression coefficient *β* = 0.52, *P* < 0.001), while psychotherapy experience was associated with lower vividness (*b* = −1.45, *P* = 0.008), intrusiveness (*b* = −1.33, *P* = 0.004) and craving intensity (*b* = −1.56, *P* = 0.010).

**Conclusions:**

Substance‐related memory intrusions in people with cocaine use disorder are distinct cognitive‐affective events that often occur independently of craving but are closely linked to its intensity, particularly when experienced as vivid and emotionally charged. Targeting these features through behavioural or pharmacological interventions may help mitigate craving‐driven distress and impulsive use‐related behaviour.

## INTRODUCTION

Cocaine use poses a critical global health concern, with approximately 22 million users world‐wide, a number that continues to grow alongside increasing supply and demand [1]. Among users, 15% to 21% are at risk of developing a cocaine use disorder (CUD), a condition marked by compulsive cocaine‐seeking behaviour despite severe psychosocial and health‐related consequences [2, 3]. Central to both the diagnosis and maintenance of CUD is craving, defined as an intense urge or desire to use the substance [4, 5]. Exposure to substance‐related cues, emotional states (negative and positive), low self‐efficacy and current abstinence have been repeatedly associated with heightened levels of cocaine craving [6, 7, 8, 9, 10, 11]. Accordingly, craving is recognized as an important promoter of substance use and of relapse [4, 11, 12, 13]. Despite advances in psychotherapeutic approaches to reduce craving and prevent relapse, high dropout rates and limited long‐term efficacy remain significant challenges [14, 15, 16].

Although craving and conditioned substance‐related cues have been extensively studied, the role of intermediary memory‐related processes remains underexplored. Theoretical models and emerging evidence from behavioural and neurobiological research suggest that maladaptive memory processes may contribute to the persistence and relapse risk in addictive behaviours [17, 18, 19]. Within this framework, these processes have been discussed under the broader concept of ‘addiction memory’, referring to the long‐term encoding of substance‐related experiences and their influence on substance‐seeking behaviour [20]. Intrusive recollections of past substance use and related cues may represent an explicit expression of addiction memory, arising spontaneously, involuntarily and without deliberate intent in daily life. These intrusions may vividly re‐evoke multi‐sensory aspects of prior cocaine use experiences, including visual or auditory imagery, bodily sensations, emotions or even gustatory and olfactory impressions [21, 22, 23].

However, although the idea of substance‐related intrusions is theoretically compelling, empirical evidence in humans remains sparse—particularly with respect to their frequency, phenomenology and associated cognitive and behavioural responses in daily life. This gap is especially striking in CUD, where craving and psychological symptoms typically outweigh physiological withdrawal [24]. Yet, systematic investigations of substance‐related intrusions in CUD are virtually absent, despite preliminary support in alcohol use disorders [25, 26].

Moreover, it remains unclear whether intrusions and craving represent overlapping or distinct cognitive phenomena. The elaborated intrusion (EI) model of craving proposed by May *et al*. [23, 27] provides a cognitive framework for how intrusions may give rise to craving. According to the model, intrusive substance‐related thoughts trigger vivid mental imagery, which—although sometimes initially pleasurable—heightens the perceived sense of lack, thereby intensifying desire and motivating substance‐seeking behaviour [23, 27]. Still, few studies have empirically examined these proposed mechanisms or investigated the phenomenology of substance‐related intrusions in naturalistic settings.

This study seeks to address these gaps by using ecological momentary assessment (EMA) to examine the dissociability of cocaine‐related intrusions from craving in individuals with CUD. By exploiting the unique advantages of EMA via smartphone‐based sampling—namely its high ecological validity and high‐density, real‐time data collection—we aimed to examine substance‐related intrusions in CUD in everyday life. We hypothesized that intrusions can occur with and without craving, with participants able to distinguish between pure intrusions, intrusions with craving and pure craving episodes. We expected that specific characteristics of intrusions, such as their vividness, intrusiveness and modality, will be associated with craving intensity and likelihood. We further hypothesized that negative preceding affective and physiological states (e.g. feeling unsafe, angry or tired) and certain trigger types will increase craving intensity and likelihood. Craving intensity and likelihood were also expected to relate to distinct emotional, physiological and cognitive‐behavioural responses, with craving‐related episodes associated with greater distress, fear and loss of control than those involving intrusions alone. Finally, we expected the severity of CUD to be associated with the frequency and type of episodes, craving intensity, intrusion characteristics and the distress, fear and loss of control associated with the episodes.

With this study, we aim to refine the understanding of CUD by highlighting the role of intrusive substance‐related memories and their emotional impact in maintaining craving and continued use. By targeting these memory‐based mechanisms, future interventions may more effectively prevent relapse and improve long‐term outcomes.

## METHODS

### Design and participants

Study participants were recruited from April 2022 to April 2023 via online advertisements, flyers in psychiatric institutions and public postings. Inclusion criteria encompassed an age between 18 and 60 years old, proficiency in German language, smartphone use, a current diagnosis of mild, moderate or severe CUD according to Diagnostic and Statistical Manual of Mental Disorders, fifth edition (DSM‐5), regular cocaine use in the past 12 months and current experiences of cocaine‐related intrusions or craving. Exclusion criteria included diagnoses of schizophrenia, bipolar disorder, autism spectrum disorder, a current post‐traumatic stress disorder (PTSD) diagnosis and a current use disorder for substances other than nicotine, moderate alcohol or moderate cannabis.

The study design consisted of a baseline visit, a 14‐day EMA monitoring period and a follow‐up phone interview. The baseline visit included demographic and clinical assessments, a training on using the smartphone ecological momentary assessment (SEMA^3^) app [28] and a standardized instruction on EMA items. The follow‐up interview assessed app usability and potential influences on reporting behaviour, such as unusual life events.

The study was approved by the Ethics Committee of the Faculty of Arts and Social Sciences, University of Zurich (no. 22.2.19) and conducted in accordance with the Declaration of Helsinki. Participants provided written informed consent and received CHF 100, with a bonus of CHF 20 for compliance rates above 75%, measured by responses to twice‐daily reminder prompts.

### Measures

#### Baseline clinical and substance use assessment

Psychiatric diagnoses were assessed by trained examiners supervised by psychologists using the Structured Clinical Interview for DSM‐5 Axis I Disorders [29]. Cocaine use history was evaluated with questions adapted from the structured Interview for Psychotropic Drug Consumption [30]. Long‐term cognitive features of cocaine craving and acute cocaine craving at baseline were assessed with the Obsessive Compulsive Cocaine Use Scale (OCCUS) [31] and the brief Cocaine Craving Questionnaire (CCQ‐Brief) [32]. In active smokers, the Fagerström Test for Nicotine Dependence was applied [33]. Additional self‐reported clinical scores included the (i) Beck Depression Inventory II (BDI‐II) [34], (ii) Attention‐Deficit/Hyperactivity Disorder Self‐Rating Scale (ADHD‐SR) [35] and (iii) Global Assessment of Functioning (GAF) scale [36]. Participants were also asked about current or past psychotherapy experiences [yes/no (Y/N)] and whether intrusions were addressed in this context (Y/N).

#### EMA

Participants were instructed and used the SEMA^3^ app to report cocaine‐related intrusion episodes—defined as sudden, involuntary memories or thoughts related to cocaine use—and/or cravings over 14 days. Participants used their own smartphones to reduce participant burden and facilitate compliance. All individuals screened for eligibility owned a smartphone, and no participants were excluded because of lack of device access.

The design was event‐based, with participants instructed to initiate an entry whenever an intrusion or craving occurred or as soon as possible thereafter. With each entry, participants could specify the type of episode by choosing between ‘pure intrusion’, ‘intrusion with subsequent craving’, ‘intrusion with simultaneous craving’ and ‘pure craving’. When craving was reported, its intensity was rated (scale from 0 to 10). Based on the intrusion questionnaire from Hackmann *et al*. [37], reported episodes were further detailed with information on intrusion vividness and intrusiveness (both 0–10) and modality (multiple‐choice options, e.g. movie‐like, emotional, bodily sensation). Participants further reported intrusion trigger types (multiple‐choice), preceding affective and physiological states (each 0–10) and cognitive‐behavioural, emotional and physiological responses (multiple‐choice). Furthermore, they rated intrusion‐related levels of distress, fear and loss of control (0–10). A full list of detailed response options is provided in Supporting information Section S1. A twice‐daily time‐related prompt served as a reminder to log any unreported episodes.

#### Statistical analysis

Data pre‐processing is described in detail in Section S1. EMA data were analysed using two‐level multi‐level models accounting for repeated observations (level 1) nested within participants (level 2). Continuous level‐1 predictors were participant‐mean centred and level‐2 predictors were grand‐mean centred [38]. All models included random intercepts for participants. Random slopes were retained if they improved model fit (likelihood ratio tests) without convergence issues. Linear regression models were used, for participant‐level outcomes.

Craving intensity included zero‐values from pure intrusion episodes without craving, which were treated as meaningful data points. To test the robustness of findings, sensitivity analyses were conducted using dichotomized craving likelihood models (0 = no craving, 1 = any craving). For binary‐coded multiple‐choice response variables (e.g. intrusion triggers, modalities, responses), descriptive mean ratios (%) were calculated by averaging, across participants, the proportion of episodes of a given type in which a specific response option was reported. Low‐frequency options (<10%) were excluded from multi‐variable models to ensure model stability. Models on intrusion vividness, intrusiveness and modalities included only episodes with reported intrusions.

Cocaine use severity (CUS) was computed as a standardized composite z‐score of log‐transformed weekly cocaine use, duration of use and the OCCUS total score, following Vonmoos *et al*. [39]. Psychotherapy experience was additionally included as a binary exploratory predictor. CUS‐only models and individual CUS components are reported in the Supporting information. Exploratory analyses also examined participant compliance and potential discrepancies between the number of episodes reported during EMA and those estimated at baseline.

All models were bootstrapped to ensure robustness of estimates. Bootstrapped results closely aligned with original estimates and are available on the Open Science Framework (OSF). In multi‐level models, coefficients reflect centred predictors, whereas in linear regression models, standardized coefficients are reported. Inferential statistics were two‐tailed with α = 0.05. False discovery rate (FDR) correction was applied to omnibus hypothesis tests [40], and corrected *P*‐values are indicated. For multi‐level models with level‐1 predictors, R^2^ (marginal/conditional) and intraclass correlation values are reported in result tables (detailed description in Supporting information Section S2).

Analyses were conducted in R (version 4.4.3). The analysis plan, including all hypotheses, was pre‐registered on OSF on 27 September 2024 (https://doi.org/10.17605/OSF.IO/E782R). Analysis data, R code and full results are accessible via the associated OSF sub‐project (https://doi.org/10.17605/OSF.IO/YAH8F) [41].

## RESULTS

### Demographic, clinical and substance use characteristics

*N* = 47 participants with CUD were initially recruited and *n* = 43 were included in the final statistical analysis. Four participants were excluded because of psychotic symptoms (*n* = 1), lack of data entry and non‐responsiveness to prompts (*n* = 2) or dropout after screening (*n* = 1). Table 1 presents descriptive statistics for demographic, clinical and substance use characteristics of the included participants. In participants that were abstinent or had significantly reduced their cocaine use at the time of assessment, cocaine use data before abstinence/reduction were used for estimating CUS. Fifteen participants stated that they had had previous or current psychotherapy, of which 11 reported intrusions had been addressed in psychotherapy.

**TABLE 1:** Demographic, clinical and substance use characteristics.

	Cocaine users (*n* = 43)
Sex *n* (female/male)	7/36 (16.3% female)
Age (years)	29.65 (7.87), range 18–59
Years of education	15.38 (3.52)
ADHD‐SR total score (0–54)	19.05 (10.26)
BDI‐II total score (0–63)	13.88 (8.97)
GAF score (0–100)	62.47 (9.49)
Psychotherapy experience *n* (y/n)	15/28
Familiar with concept ‘intrusions’ *n* (y/n)	11/4
Cocaine use and craving	
Cocaine use disorder severity	
Mild *n*	1
Moderate *n*	8
Severe *n*	34
Age of onset of use	21.55 (5.47)
Duration of cocaine use (years)	8.10 (5.83)
Maximum of use within 1 day (g)	3.14 (1.78)
Current cocaine use (*n* = 33)	
Frequency of use (occasions per week)	1.85 (1.85)
Dose per week (g)	1.25 (1.42)
Abstinent or significantly reduced cocaine use (*n* = 10)	
Current frequency (occasions per week)	0.26 (0.54)
Current dose per week (g)	0.22 (0.42)
Earlier frequency (occasions per week)	4.10 (2.63)
Earlier dose per week (g)	6.22 (8.40)
Duration of abstinence/use reduction (days)	59.45 (80.73)
OCCUS total score (0–54)	19.65 (7.37)
CCQ mean score [1–7]	5.21 (1.44)
Use of further substances	
Smoking status *n* (y/n)	38/5
Fagerström score (nicotine)	3.26 (2.11)
Alcohol use disorder *n* (y/n)	14/29
Mild *n*	8/14
Moderate *n*	6/14
Cannabis use disorder *n* (y/n)	7/38
Mild *n*	6/7
Moderate *n*	1/7
Compliance	
Total no. of reported episodes (14 days of EMA)	8.37 (5.79)
No. of episodes during 14 days estimated at baseline	25.42 (23.45)
Average discrepancy (baseline–EMA)	18.53 (18.96)
Compliance measured by responses to daily reminder prompts (%)	70.58 (24.1)

*Note*: Values reflect mean and SDs.

Abbreviations: ADHD‐SR, ADHD Self‐Rating Scale; BDI‐II, Beck Depression Inventory II; CCQ, Cocaine Craving Questionnaire; EMA, ecological momentary assessment; GAF, Global Assessment of Functioning scale; OCCUS, Obsessive Compulsive Cocaine Use Scale; y/n, yes/no.

### Distribution of intrusion episodes across participants

**FIGURE 1:**
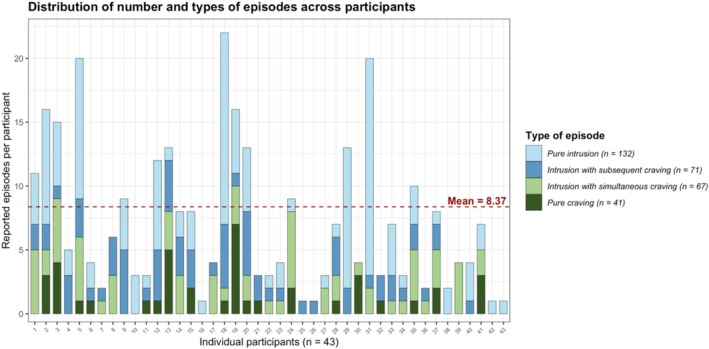
Distribution of number and types of episodes by each participant. The dashed line indicates the mean number of reported episodes across participants.

Over the 14‐day monitoring period, participants initiated a total of 360 entries [mean (M) = 8.37, SD = 5.79, range = 2–22]. After excluding 49 early‐stage incomplete entries, 311 complete entries were analysed. Pure intrusions were the most frequently reported episode type (*n* = 132, 42.4%), followed by intrusions with subsequent craving (*n* = 71, 22.8%), intrusions with simultaneous craving (*n* = 67, 21.5%) and pure craving episodes (*n* = 41, 13.2%). Figure 1 illustrates intra‐ and inter‐individual variability in the frequency and distribution of episode types. Exploratory analyses of craving intensity across craving‐related episode types revealed no significant differences (all *P* > 0.05) (Table S1).

### Intrusion characteristics and craving outcomes

**FIGURE 2:**
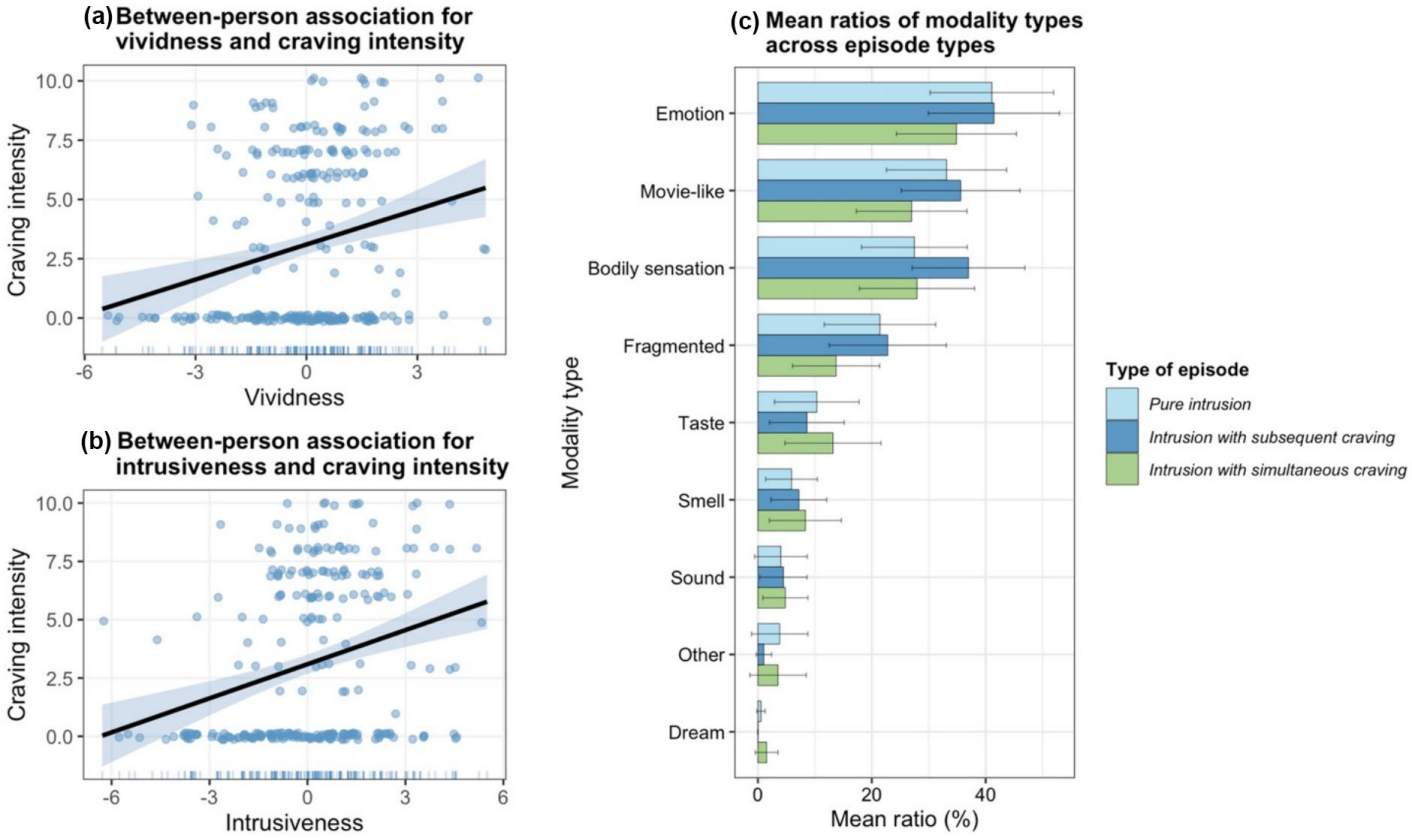
(a) Between‐person association between vividness and craving intensity. (b) Between‐person association between intrusiveness and craving intensity. (c) Mean ratios of different intrusion modality types for different episode types, averaged across participants. Error bars represent 95% CIs.

The most frequently reported intrusion modalities across episode types were emotions, bodily sensations and movie‐like memories and fragmented memories. In Figure 2(c), mean ratios for all modalities, stratified by episode type, are presented. Bodily sensations were particularly associated with higher craving intensity and likelihood (Table 2). On average, intrusion vividness was rated at M = 6.01 (SD = 2.50) and intrusiveness at M = 6.07 (SD = 2.56). Separate mixed‐effects models revealed that, across participants, intrusions rated as more vivid and more intrusive were significantly associated with higher craving intensity and greater craving likelihood [Table 2; Figure 2(a,b)].

**TABLE 2:** Associations between intrusion characteristics, triggers and responses and craving.

Intrusion characteristics	Craving intensity (0–10)			Craving likelihood (0/1)		
	Est.	CI	*P*	OR	CI	*P*
Modalities						
Emotion	0.57	[−0.32 to 1.46]	0.208	1.40	[0.74–2.65]	0.294
Bodily sensation	**1.11**	**[0.23–1.99]**	**0.013**	**2.26**	**[1.23–4.15]**	**0.009**
Fragmented	−0.29	[−1.32 to 0.74]	0.576	0.69	[0.34–1.40]	0.305
Movie‐like	−0.03	[−0.94 to 0.88]	0.949	0.93	[0.50–1.72]	0.811
Taste	0.78	[−0.48 to 2.03]	0.225	1.64	[0.69–3.91]	0.261
Smell	−0.57	[−2.00 to 0.86]	0.435	0.95	[0.36–2.54]	0.919
No. of obs.	270^a^			270^a^		
R^2^ marginal/conditional	0.046/0.208			0.071/0.270		
ICC	0.17			0.21		
Vividness	**0.53**	**[0.21–0.86]**	**0.002**	**1.53**	**[1.27–1.85]**	**<0.001**
Number of obs.	270^a^			270^a^		
R^2^ marginal/conditional	0.073/0.245			0.115/0.323		
ICC	0.19			0.23		
Intrusiveness	**0.48**	**[0.30–0.67]**	**<0.001**	**1.67**	**[1.39–2.01]**	**<0.001**
No. of obs.	270^a^			270^a^		
R^2^ marginal/conditional	0.078/0.233			0.195/0.423		
ICC	0.17			0.28		
Triggers						
Trigger types						
Thoughts/emotions	**0.98**	**[0.13–1.82]**	**0.024**	**2.34**	**[1.25–4.38]**	**0.008**
Place/environment/situation	0.76	[−0.08 to 1.61]	0.077	**1.94**	**[1.06–3.56]**	**0.031**
People	0.36	[−0.57 to 1.29]	0.446	1.51	[0.77–2.94]	0.228
Conversations	0.05	[−0.98 to 1.08]	0.926	1.27	[0.60–2.68]	0.537
Perceptions	−0.48	[−1.71 to 0.74]	0.439	0.94	[0.40–2.19]	0.884
None	−1.00	[−2.34 to 0.34]	0.142	0.47	[0.19–1.19]	0.114
No. of obs.	311			311		
R^2^ marginal/conditional	0.053/0.220			0.108/0.373		
ICC	0.18			0.30		
Affective state						
Comfort	0.03	[−0.19 to 0.25]	0.784	1.07	[0.92–1.23]	0.396
Relaxation	−0.04	[−0.26 to 0.17]	0.678	0.88	[0.76–1.02]	0.092
Safety	**−0.24**	**[−0.48 to 0.00]**	**0.048**	0.90	[0.77–1.05]	0.191
Anger	0.17	[−0.04 to 0.38]	0.110	1.05	[0.91–1.21]	0.482
No. of obs.	310			310		
R^2^ marginal/conditional	0.032/0.190			0.033/0.251		
ICC	0.16			0.23		
Physiological state						
Tiredness	−0.05	[−0.24 to 0.14]	0.608	1.07	[0.94–1.22]	0.301
Energy levels	0.13	[−0.08 to 0.35]	0.229	**1.18**	**[1.02–1.36]**	**0.030**
Hunger	0.08	[−0.08 to 0.23]	0.327	1.03	[0.93–1.14]	0.616
Sexual arousal	0.15	[−0.04 to 0.34]	0.114	**1.15**	**[1.02–1.31]**	**0.026**
No. of obs.	310			310		
R^2^ marginal/conditional	0.020/0.176			0.038/0.259		
ICC	0.16			0.23		
Responses						
Cognitive‐behavioural responses						
Distraction	−0.08	[−0.90 to 0.74]	0.850	0.84	[0.45–1.57]	0.590
Rumination	−0.24	[−1.12 to 0.64]	0.589	**0.47**	**[0.24–0.93]**	**0.031**
Suppression	0.02	[−0.85 to 0.89]	0.964	0.71	[0.37–1.33]	0.282
Conscious engagement	−0.05	[−1.19 to 1.09]	0.929	0.46	[0.19–1.11]	0.083
Social contact	−0.46	[−1.10 to 0.68]	0.426	1.10	[0.46–2.61]	0.830
Obtain cocaine	**3.44**	**[2.31–4.57]**	**<0.001**	**19.90**	**[5.12–77.39]**	**<0.001**
Substance use	**1.12**	**[0.19–2.05]**	**0.018**	1.80	[0.87–3.73]	0.116
No. of obs.	311			311		
R^2^ marginal/conditional	0.154/0.286			0.285/0.455		
ICC	0.16			0.24		
Emotional responses						
Guilt	**1.26**	**[0.24–2.28]**	**0.016**	**2.27**	**[1.06–4.88]**	**0.035**
Shame	−0.59	[−1.60 to 0.43]	0.258	0.95	[0.46–1.98]	0.897
Helplessness	−0.16	[−1.16 to 0.83]	0.747	0.81	[0.40–1.65]	0.562
Annoyance	−0.27	[−1.23 to 0.70]	0.589	0.96	[0.48–1.93]	0.913
Irritability	**1.34**	**[0.20–2.47]**	**0.021**	**2.40**	**[1.04–5.53]**	**0.041**
Anticipatory pleasure	**1.40**	**[0.43–2.38]**	**0.005**	**3.52**	**[1.70–7.30]**	**0.001**
Do not know	**−1.35**	**[−2.53 to −0.17]**	**0.025**	0.70	[0.30–1.62]	0.402
No. of obs.	311			311		
R^2^ marginal/conditional	0.106/0.269			0.126/0.337		
ICC	0.18			0.24		
Physiological responses						
Inner restlessness	1.12	[−0.02 to 2.27]	0.055	**2.41**	**[1.08–5.39]**	**0.033**
Motor restlessness	0.48	[−0.81 to 1.78]	0.465	1.31	[0.52–3.32]	0.570
Tension	**0.97**	**[0.04–1.90]**	**0.041**	1.47	[0.76–2.84]	0.256
Racing heart	1.30	[−0.08 to 2.67]	0.064	1.59	[0.57–4.43]	0.380
None	−0.06	[−1.47 to 1.35]	0.932	0.76	[0.29–2.00]	0.584
No. of obs.	311			311		
R^2^ marginal/conditional	0.073/0.238			0.090/0.318		
ICC	0.18			0.25		

*Note*: Values reflect regression coefficients or ORs, 95% CI and *P*‐values. Bold values indicate statistical significance (*P* < 0.05). Statistical tests: linear or logistic mixed‐effects models with a random intercept for participants and additional random slopes for the linear mixed‐effects model with vividness as predictor.

Abbreviations: ICC, intraclass correlation; obs., observations.

^a^ Models based on 270 entries due to exclusion of pure craving episodes, which did not involve an intrusion.

^b^ Models based on 310 entries due to missing values in affective and physiological state variables for one observation.

### Episode triggers and craving outcomes

Across all episode types, participants most commonly identified place/environment/situation, thoughts/emotions and people as triggers for the respective episodes. Figure S1 shows mean ratios for all trigger types, stratified by episode type. Mixed‐effects models revealed that thoughts/emotions significantly predicted higher craving intensity, whereas triggers thoughts/emotions, and place/environment/situation significantly increased craving likelihood (Table 2).

Feeling more safe before an intrusion episode was associated with lower craving intensity, while affective states did not predict craving likelihood. Greater perceived energy levels and sexual arousal were positively associated with craving likelihood. However, neither craving intensity nor likelihood was predicted by tiredness or hunger (Table 2).

### Responses to episodes and craving outcomes

**FIGURE 3:**
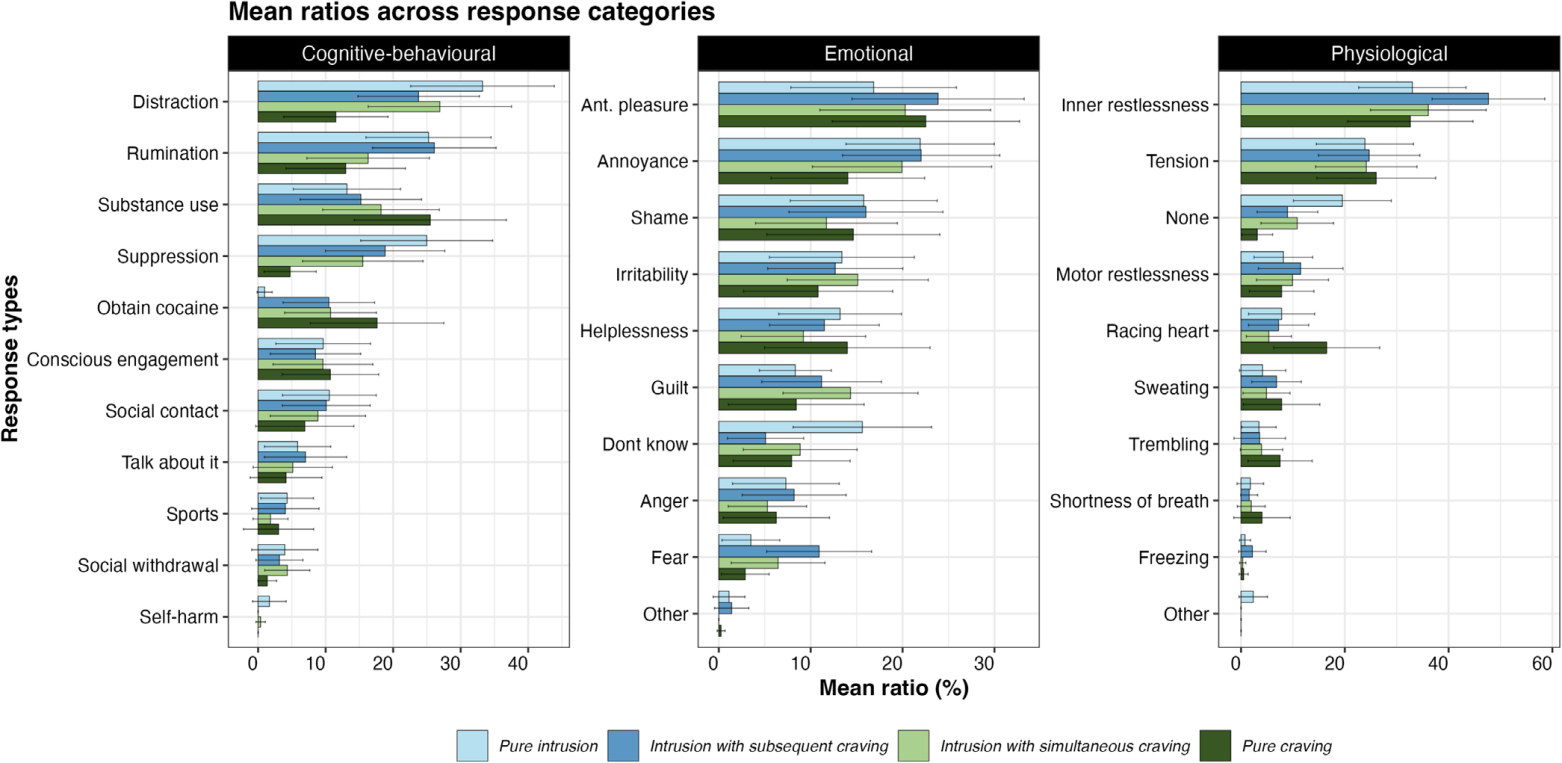
Mean ratios of (a) cognitive‐behavioural, (b) emotional and (c) physiological reaction types across episode types, averaged across participants. Error bars represent 95% CIs.

In terms of cognitive‐behavioural responses associated with episodes, distraction, rumination, substance use, suppression and attempts to obtain cocaine were most frequently reported. Substance use and obtaining cocaine were significantly associated with higher craving intensity, with the latter as well as rumination also linked to greater craving likelihood. Regarding emotional responses, participants most commonly reported feelings of anticipatory pleasure, annoyance and shame. Guilt, irritability and anticipatory pleasure positively predicted craving intensity and likelihood. Conversely, participants who reported being unable to identify an emotional response (‘do not know’) demonstrated significantly lower craving intensity. Among physiological responses, the predominantly reported tension and inner restlessness were significant predictors of craving intensity and likelihood, respectively (Table 2). Figure 3 presents mean ratios for all response types, stratified by episode type.

### Episode‐related emotional intensity

**FIGURE 4:**
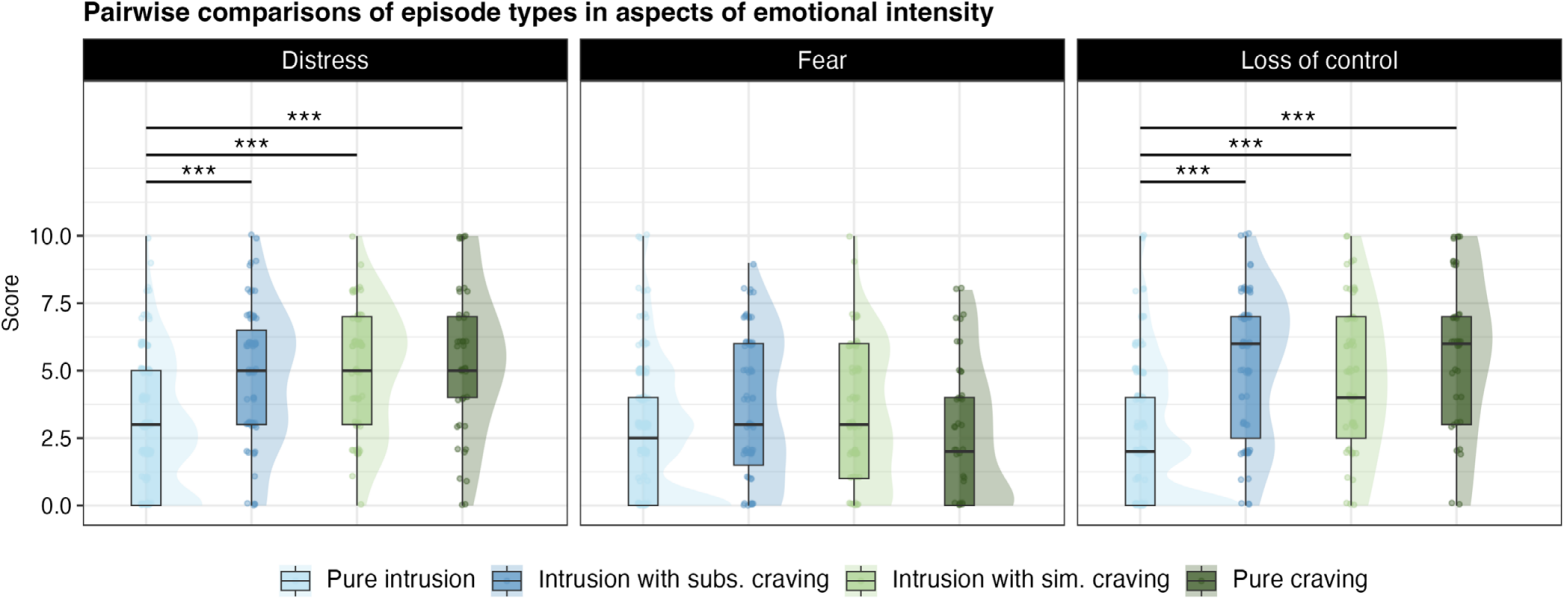
Boxplots with overlaid data points show distress, fear and loss of control scores for different episode types. Median and quartiles are displayed. Violin plots indicate data distribution. Significant group differences based on false discovery rate (FDR)‐corrected pairwise comparisons are highlighted (***P < 0.001).

For episodes involving craving, with or without reported intrusions, distress and loss of control were significantly higher than for pure intrusions. Fear was generally low across all episode types, with no significant differences observed (Table 3, Figure 4). Exploratively, distress and loss of control were significantly associated with craving intensity and likelihood across episode types (all *P* ≤ 0.001) (Table S2).

**TABLE 3:** Pairwise comparisons of episode types in distress, fear and loss of control.

Episode types‐contrasts	Distress			Fear			Loss of control		
	Est.	CI	*P*_FDR_	Est.	CI	*P*_FDR_	Est.	CI	*P*_FDR_
Type 1 versus Type 2	**−1.52**	**[−2.39 to −0.66]**	**<0.001**	−0.54	[−1.39 to 0.31]	0.283	**−2.48**	**[−3.49 to −1.48]**	**<0.001**
Type 1 versus Type 3	**−2.08**	**[−3.01 to −1.15]**	**<0.001**	−0.76	[−1.67 to 0.16]	0.178	**−2.41**	**[−3.49 to −1.33]**	**<0.001**
Type 1 versus Type 4	**−2.17**	**[−3.26 to −1.07]**	**<0.001**	−0.20	[−1.28 to 0.88]	0.622	**−3.22**	**[−4.49 to −1.95]**	**<0.001**
Type 2 versus Type 3	−0.55	[−1.57 to 0.46]	0.177	−0.22	[−1.21 to 0.78]	0.622	0.07	[−1.10 to 1.25]	0.871
Type 2 versus Type 4	−0.64	[−1.81 to 0.53]	0.177	0.34	[−0.81 to 1.49]	0.622	−0.74	[−2.09 to 0.62]	0.181
Type 3 versus Type 4	−0.09	[−1.26 to 1.08]	0.838	0.55	[−0.60 to 1.70]	0.403	−0.81	[−2.17 to 0.55]	0.173
No. of observations	311			311			311		
R^2^ marginal/conditional	0.125/0.424			0.014/0.404			0.184/0.395		
ICC	0.34			0.40			0.26		

*Note*: Values reflect regression coefficients, 95% CI and FDR‐corrected *P*‐values. Bold values indicate statistical significance (*P* < 0.05). Statistical tests: linear mixed‐effects models with a random intercept for participants, with subsequent pairwise comparisons.

Abbreviations: FDR, false discovery rate; ICC, intraclass correlation; Type 1, pure intrusion; Type 2, intrusion with subsequent craving; Type 3, intrusion with simultaneous craving; Type 4, pure craving.

### Associations with CUS and psychotherapy experience

**FIGURE 5:**
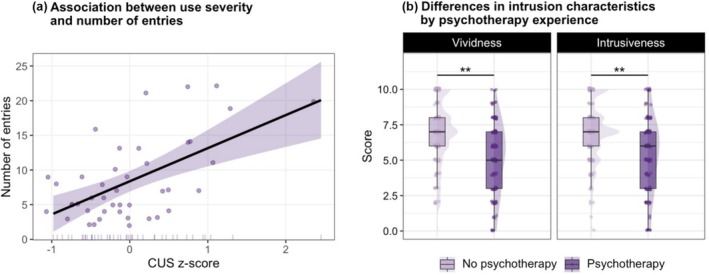
(a) Between‐person association between cocaine use severity (CUS) and number of ecological momentary assessment (EMA) entries. (b) Boxplots with overlaid data points show vividness and intrusiveness scores for participants with or without psychotherapy experience. Median and quartiles are displayed. Violin plots indicate data distribution. Significant group differences are highlighted (**P < 0.01).

Higher CUS was associated with a greater number of reported episodes while controlling for psychotherapy experience [Figure 5(a); Table 4). Those with psychotherapy experience reported more pure intrusions, although this association did not survive FDR‐correction (*P*_FDR_ = 0.056). Psychotherapy experience was also significantly associated with lower intrusion vividness and intrusiveness [Figure 5(b)] as well as lower craving intensity, whereas CUS had no significant effects on these characteristics when controlling for psychotherapy experience. Neither CUS nor psychotherapy experience significantly predicted distress, fear or loss of control associated with episodes (Table 4). Estimates for CUS‐only models and individual components of CUS are displayed in Tables S3 and S4.

**TABLE 4:** Effects of CUS and psychotherapy experience*.*

	Predictors					
	CUS z‐score			Psychotherapy experience (no/yes)		
	Est.	CI	*P*_*(FDR)*_	Est.	CI	*P*_*(FDR)*_
Total no. of entries	**0.52**	**[0.26 to 0.79]**	**<0.001**	0.19	[−0.08 to 0.45]	0.159
Ratios of episode types						
Type 1	0.24	[−0.05 to 0.53]	0.389	0.37	[0.08–0.65]	0.056
Type 2	0.09	[−0.24 to 0.42]	0.688	−0.09	[−0.41 to 0.24]	0.599
Type 3	−0.10	[−0.43 to 0.22]	0.688	−0.09	[−0.41 to 0.24]	0.599
Type 4	0.06	[−0.25 to 0.38]	0.688	−0.29	[−0.61 to 0.03]	0.144
Vividness	−0.04	[−0.68 to 0.76]	0.915	**−1.45**	**[−2.53 to −0.38]**	**0.008**
Intrusiveness	−0.04	[−0.62 to 0.55]	0.907	**−1.33**	**[−2.24 to −0.42]**	**0.004**
Distress	−0.04	[−0.81 to 0.74]	0.923	−0.43	[−1.59 to 0.73]	0.466
Fear	−0.09	[−0.91 to 0.73]	0.826	0.21	[−1.01 to 1.43]	0.735
Loss of control	−0.05	[−0.83 to 0.73]	0.895	−0.63	[−1.81 to 0.56]	0.299
Craving intensity	−0.40	[−1.16 to 0.35]	0.292	**−1.56**	**[−2.74 to −0.38]**	**0.010**

*Note*: Values reflect regression coefficients, 95% CI and *P*‐values. *P*‐values for outcomes ‘Ratios of episode types’ were FDR‐corrected within each predictor. Bold values indicate statistical significance (*P* < 0.05). Statistical tests: linear regression models or linear mixed‐effects models with a random intercept for participants.

Abbreviations: CUS, cocaine use severity; FDR, false discovery rate.

### Compliance

Participants responded to reminder prompts with a median compliance rate of 82.8% (M = 70.6%). On average, participants overestimated the frequency of such episodes at baseline compared to what was recorded via EMA, with a mean discrepancy of 18.53 entries (Table 1). Although compliance was not significantly associated with the total number of reported episodes (β = −0.28, CI = −0.58 to 0.02, *P* = 0.07), higher compliance was related to smaller discrepancies between baseline estimates and EMA‐entries (β = −0.32, CI = −0.62 to −0.03, *P* = 0.033). Table S5 depicts non‐significant effects of CUS and psychotherapy experience on compliance and discrepancy.

## DISCUSSION

This study provides a first ecological validation of substance‐related memory intrusions in individuals with CUD. Over a 2‐week EMA period, we captured the frequency, phenomenology and emotional impact of these intrusions in daily life. Our findings support their relevance as distinct and accessible cognitive‐affective events—often occurring independently of craving—and align with theoretical frameworks such as the EI model. Beyond distinguishing intrusions from craving, we offer novel insights into the vivid, multi‐sensory and emotionally charged nature of these experiences, their associations with craving intensity and cognitive‐behavioural responses and how psychotherapy experience moderated their vividness and intrusiveness.

Over 2 weeks, all participants reported at least one episode involving an intrusion, while none reported only pure craving, highlighting the general accessibility of intrusions in this population. Greater CUS was associated with a higher number of intrusion entries, and there was substantial variability in episode types both within and across participants. Intrusions frequently occurred in the absence of craving, reinforcing the EI model's conceptualization of intrusions as distinct events [23, 27, 42].

Participants described a range of intrusion modalities, most frequently reporting fragmented or movie‐like memories, emotions, bodily sensations and the taste of cocaine, highlighting their multi‐sensory and emotionally rich nature. Bodily sensations were particularly predictive of craving intensity and likelihood, likely because they mimic the sensory experience of cocaine use and enhance vividness. The frequent reporting of movie‐like memories, reminiscent of flashback‐like intrusions seen in PTSD [43], further illustrates the explicit and perceptually rich nature of these events in CUD.

Beyond modality, intrusion vividness and intrusiveness were rated as moderate to high on average. Both were significantly associated with higher craving intensity and likelihood, consistent with earlier findings in alcohol users [25, 42, 44]. This may reflect the intensification of craving through elaborative processes, whereby initial intrusive images evolve into compelling multi‐sensory experiences [42]. From a neuroscientific perspective, vivid cognitive images may involve heightened activation of substance‐related memory networks or ‘wanting circuits’ [45, 46]. Interestingly, psychotherapy experience was significantly associated with lower ratings of vividness, intrusiveness and craving intensity, possibly reflecting either reduced salience of substance‐related intrusions or more effective regulation of elaborative processing.

Still, pure craving episodes without identifiable intrusions raise questions about their underlying mechanisms. One possibility is that these episodes reflect automatic cue‐reactive processes that bypass the activation of explicit memory content. Another is that intrusive memories were triggered, but remained outside of conscious awareness—too fleeting or subtle to be recognized as discrete intrusions. In some cases, these intrusions may have been ‘overjumped’, with craving emerging directly in response to contextual or internal cues, highlighting the dynamic and context‐sensitive nature of these processes. Supporting this interpretation, participants without psychotherapy experience reported a lower proportion of pure intrusions, suggesting that therapeutic experience may enhance awareness of such internal events, although this association did not survive correction.

Turning to the contextual conditions that may precipitate such intrusions, the broad category of thoughts and feelings emerged as the most common intrusion trigger and was significantly associated with higher craving intensity and likelihood, consistent with prior research identifying both substance‐related thoughts and emotional states as critical craving triggers [6, 7, 10]. Moreover, feelings of insecurity, heightened energy and sexual arousal were particularly associated with heightened craving. Aligning with the EI model, emotional vulnerability or high arousal states may amplify elaborative processes, intensifying the need for relief or stimulation [23, 27]. Therefore, monitoring and addressing internal states such as negative affect, insecurity or heightened arousal may be crucial for preventing craving escalation.

The responses to episodes reported provided additional insights into the craving cycle. Episodes involving craving, especially pure craving episodes, were predominantly associated with impulsive responses, such as substance use and attempts to obtain cocaine. Meanwhile, episodes involving intrusions were more likely to elicit cognitive, although not necessarily functional, strategies such as rumination and distraction or efforts at suppression. These findings support the notion that craving activates motivational pathways, prompting immediate action, whereas intrusions may trigger cognitive engagement in an effort to control their impact [42]. Importantly, the overall relatively low use of functional strategies, like conscious engagement with or discussion of the intrusions, indicates a gap in adaptive coping skills or therapeutic strategies.

The emotional intensity of craving‐related episodes was evident in higher ratings of distress and loss of control compared to pure intrusions. This aligns with theories of craving‐related dissonance, where the urge to use conflicts with the desire to quit, fostering feelings of helplessness. These feelings may be pronounced in individuals with low self‐efficacy [9, 47]. Alternatively, distressing intrusions, such as vivid or disturbing recollections of past substance use, may amplify craving, consistent with findings by Berry *et al*. [48], who reported that intrusions provoking negative affect were strongly linked to craving frequency and intensity. Emotions such as guilt and irritability were positively associated with craving intensity and likelihood, potentially reflecting internalized stigma or frustration with the craving process. Conversely, joyful anticipation highlights the positive reinforcement mechanisms underlying craving [7]. Finally, physiological responses, such as tension and inner restlessness, also predicted craving outcomes, further emphasizing the embodied nature of the craving experience.

These findings highlight important clinical implications. Our results suggest that specific features of intrusions, particularly their vividness and intrusiveness, are closely linked to craving, indicating potential treatment targets. As previously proposed by May and colleagues [27], behavioural approaches such as mindfulness‐based strategies may help individuals recognize substance‐related intrusions as transient cognitive events, reducing emotional engagement and their elaboration into cravings [42]. Techniques like body scanning, which engage visuospatial and executive working memory, could be particularly effective in mitigating the vividness of intrusions. Furthermore, pharmacological approaches targeting memory reconsolidation also hold promise. By disrupting the stabilization of reactivated substance‐related memories, these interventions may weaken the salience of intrusions and reduce their associated emotional burden, complementing behavioural strategies to manage craving cycles [49, 50, 51, 52, 53, 54, 55].

This study has several limitations that warrant consideration. First, although our findings suggest that intrusions may often precede craving, the cyclical nature of these processes and the lack of an option to report craving with subsequent intrusions limit conclusions about temporal order. The absence of fine‐grained temporal modelling further restricts causal interpretation, underscoring the value of real‐time or longitudinal approaches in future research. Second, the moderate sample size and some incomplete EMA data may have limited statistical power and generalizability, particularly for subgroup or interaction analyses. Although the sex distribution reflects typical patterns in substance‐using populations [56], the low proportion of female participants precluded analysis of gender effects, which may be relevant to craving dynamics [10, 57]. Moreover, recruitment relied primarily on convenience sampling, so the representativeness of the sample relative to the broader CUD population is uncertain. Therefore, the frequency and phenomenology of intrusions and craving observed here may not fully generalize to all individuals with CUD. Third, cocaine use measures relied on self‐reports, which can be affected by recall and social desirability biases [58]. Future studies should incorporate objective measures, such as hair analyses, to enhance reliability. Despite high overall compliance, discrepancies between baseline estimates and EMA‐reported episodes suggest potential under‐reporting—possibly because of missed fleeting intrusions or entries in highly triggering situations [59]. Although the event‐based design helped reduce participant burden, it may have missed episodes. Integrating more time‐based prompts in future studies could improve data completeness. Finally, we did not directly assess interoceptive awareness, which may influence the recognition of intrusions or cravings, nor did we examine the effect of comorbid conditions such as ADHD or depression, which could shape these experiences. These variables should be addressed in future research. Despite these limitations, the study's use of EMA in a naturalistic setting provides novel and ecologically valid insights into the daily‐life dynamics of intrusions and craving in CUD.

Together, this study offers the first ecologically valid evidence of cocaine‐related intrusions as frequent, distinct and impactful cognitive‐affective events in CUD. Their dissociability from craving, yet strong predictive value for craving intensity when more vivid and intrusive, underscores their clinical relevance. Our findings position intrusions as key phenomena—transdiagnostic in nature and a key target in substance use disorders. Interventions that identify and modify intrusions, thereby reducing their salience and elaboration, may interrupt craving cycles before they escalate and could offer powerful avenues for relapse prevention and sustained behaviour change.

## AUTHOR CONTRIBUTIONS

**Amelie Zacher:** Conceptualization (lead); data curation (lead); formal analysis (lead); investigation (lead); methodology (equal); project administration (lead); visualization (lead); writing—original draft (lead). **Lina Dietiker:** Conceptualization (equal); data curation (supporting); investigation (lead); methodology (equal); project administration (lead); writing—review and editing (equal). **Victoria Häffner:** Investigation (equal); project administration (supporting); writing—review and editing (supporting). **Francesco Bavato:** Conceptualization (equal); methodology (supporting); writing—review and editing (equal). **Birgit Kleim:** Conceptualization (equal); funding acquisition (lead); methodology (equal); project administration (supporting); supervision (lead); writing—review and editing (equal). **Boris B. Quednow:** Conceptualization (lead); funding acquisition (lead); methodology (equal); project administration (supporting); supervision (lead); writing—review and editing (equal).

## DECLARATION OF INTERESTS

None.

## Supporting information


**Table S1.** Pairwise comparisons of craving‐related episode types in craving intensity.
**Table S2.** Distress, fear and loss of control and craving outcomes.
**Table S3.** Associations between CUS and episode characteristics, craving, and compliance.
**Table S4.** Associations between individual components of CUS and episode characteristics, craving, and compliance.
**Table S5.** Associations between CUS and psychotherapy, and compliance rate and discrepancy.
**Figure S1.** Supplementary Information.

## Data Availability

The data that support the findings of this study are openly available on OSF at https://doi.org/10.17605/OSF.IO/YAH8F.
