# Supplementary material for: Substance‐related intrusive memories in cocaine use disorder are different from but associated with craving
Source: Addiction. 2026 Apr 27;121(9):2504–17. doi: 10.1111/add.70449 (PMC13486936; doi:10.1111/add.70449)
Supplement: Supplementary file 1 [file ADD-121-2504-s001.docx]

**Supplementary information**

**Substance-related intrusive memories in cocaine use disorder are different from, but associated with craving**

Amelie Zacher^1^, Lina Dietiker^2,3^, Victoria Häffner^1^, Francesco Bavato^1,4^, Birgit Kleim^2,3*^, Boris B. Quednow^1,5*^

*^1^Experimental Pharmacopsychology and Psychological Addiction Research, Department of Adult Psychiatry and Psychotherapy, University Hospital of Psychiatry Zurich, University of Zurich, Zurich, Switzerland*

*^2^Experimental Psychopathology and Psychotherapy, Department of Psychology, University of Zurich, Zurich, Switzerland*

*^3^Department of Adult Psychiatry and Psychotherapy, University Hospital of Psychiatry Zurich, University of Zurich, Zurich, Switzerland*

*^4^Center of Integrative Functional Neuroscience, Department of Medicine, Aarhus University, Denmark*

*^5^Neuroscience Centre Zurich, Joint Centre of University of Zurich and Swiss Federal Institute of Technology Zurich, Zurich, Switzerland*

Table of Contents

[Supplementary Section 1: Methods	2](#_Toc214293545)

[EMA response options	2](#_Toc214293546)

[Data pre-processing	3](#_Toc214293547)

[Supplementary Section 2: Variance decomposition in multilevel models	3](#_Toc214293548)

[Supplementary Section 3: Supplementary Tables	5](#_Toc214293549)

[Supplementary Table S1	5](#_Toc214293550)

[Supplementary Table S2	5](#_Toc214293551)

[Supplementary Table S3	6](#_Toc214293552)

[Supplementary Table S4	7](#_Toc214293553)

[Supplementary Table S5	8](#_Toc214293554)

[Supplementary Section 4	8](#_Toc214293555)

[Supplementary Figure S1	8](#_Toc214293556)

# **Supplementary Section 1: Methods**

## **EMA response options**

The EMA app used in this study allowed participants to report detailed information about their cocaine-related episodes. Participants were asked to identify the types of trigger for the intrusion (multiple-choice options: “no identifiable trigger”, “thoughts/emotions”, “conversations”, “people”, “dream”, “place/environment/situation”, “sensory perception”, “news/TV”, “other”), and to rate their preceding affective state (levels of safety, relaxation, comfort and anger) and physiological state (levels of tiredness, energy, hunger, and sexual arousal) on scales from 0 to 10. Participants were further asked to specify the vividness and intrusiveness of intrusions (both on scales from 0 to 10), as well as the modality in which it occurred (multiple-choice options: “movie-like memory”, “fragmented memory”, “dream”, “emotion”, “bodily sensation”, “sound/noise”, “smell”, “taste”, “other”). Reported episodes were rated for associated distress, fear and loss of control (0-10, with 0 = no level and 10 = very high levels). Finally, participants were asked about their responses to the episodes on cognitive-behavioural, emotional, and physiological levels, again using multiple-choice questions. They specified their cognitive-behavioural responses (options: “rumination”, “suppression”, “distraction”, “self-harm”, “sports”, “initiate social contact”, “social withdrawal”, “conscious engagement with intrusion”, “talk about it with someone”, “trying to obtain cocaine”, “substance use”, “other”), emotional responses (options: “anger”, “fear”, “guilt”, “shame”, “helplessness”, “annoyance”, “irritability”, “excitement”, “other”, “I don’t know”), and physiological responses (options: “tension”, “racing heart”, “shortness of breath”, “trembling”, “sweating”, “freezing”, “inner restlessness”, “motor restlessness”, “other”, “none”).

## **Data pre-processing**

Data pre-processing involved systematic exclusion of entries where the security question about experiencing an intrusion or craving was answered with “no”, and of 49 early-stage incomplete observations, where predictors and/or outcome variables of models were missing. Since the reason for their incompleteness could not be definitely determined and data were unlikely to be missing at random, no imputation was performed. In models examining affective or physiological states, one additional entry was excluded due to missing values in the respective variables, but was retained for all other models. Outliers were not excluded in any analyses, given their potential importance for understanding individual variability. Baseline data were complete, with no exclusions necessary.

# **Supplementary Section 2: Variance decomposition in multilevel models**

To complement the main results, intraclass correlation coefficients (ICC) and R^2^ marginal and conditional values are reported for multilevel models predicting craving intensity and likelihood, as well as for models examining distress, fear, and loss of control across episode types, providing insight into the proportion of variance explained by predictors and the extent to which the outcomes are influences by within-person versus between-person variability.

For craving intensity, ICC values ranged from 0.16 to 0.19, indicating that most variability in intensity occurred within individuals over time. Craving likelihood showed higher ICC values (0.23 to 0.30), suggesting that individual differences played a greater role in determining whether craving occurred at all. The variance explained by fixed effects alone was low across craving intensity models (R^2^ marginal = 0.02-0.15), while the full model, including random effects, explained more variance (R^2^ conditional = 0.18-0.29), reinforcing the contribution of individual differences.

For episode-related feelings of distress, fear, and loss of control, ICC values were higher (0.34, 0.40, and 0.26, respectively), indicating that these reactions were more strongly influenced by between-person differences compared to craving intensity. Fixed effects explained only a small portion of variance (R^2^ marginal = 0.01-0.18), while full models accounted for substantially more (R^2^ conditional = 0.40-0.42), highlighting the role of stable individual characteristics.

Overall, the results suggest that craving intensity is highly dynamic, while craving likelihood and episode-related feelings of distress, fear and loss of control show stronger between-person influences. The gap between R^2^ marginal and R^2^ conditional across models further supports the interplay of state-dependent and trait-like factors in craving episodes and emotional impact.

# **Supplementary Section 3: Supplementary Tables**

## **Supplementary Table S1**

*Pairwise comparisons of craving-related episode types in craving intensity*

|  | **Craving intensity (0-10)** | | |
| --- | --- | --- | --- |
|  | *Est.* | *CI* | *p_FDR_* |
| **Episode types-Contrasts** |  | | |
| Type 2 vs. Type 3 | 0.16 | [-0.99, 1.31] | 0.737 |
| Type 2 vs. Type 4 | 0.53 | [-0.79, 1.86] | 0.736 |
| Type 3 vs. Type 4 | 0.37 | [-0.94, 1.68] | 0.736 |
| Number of observations | 179 | | |
| R^2^ Marginal / Conditional | 0.005 / 0.306 | | |
| ICC | 0.30 | | |

*Note.* Values reflect regression coefficients, 95% confidence intervals and FDR-corrected *p*-values. ICC, Intra-Class-Correlation. Statistical tests: Linear mixed-effects models with a random intercept for participants, with subsequent pairwise comparisons. Type 2, Intrusion with subsequent craving; Type 3, Intrusion with simultaneous craving; Type 4, Pure craving.

## **Supplementary Table S2**

*Distress, fear and loss of control and craving outcomes*

|  | **Craving intensity (0-10)** | | | **Craving likelihood (0/1)** | | |
| --- | --- | --- | --- | --- | --- | --- |
|  | *Est.* | *CI* | *p* | *OR* | *CI* | *p* |
| *Distress* | **0.34** | **[0.14, 0.54]** | **0.001** | **1.35** | **[1.13, 1.62]** | **0.001** |
| *Fear* | -0.05 | [-0.25, 0.15] | 0.626 | 0.92 | [0.78, 1.09] | 0.347 |
| *Loss of control* | **0.43** | **[0.27, 0.58]** | **<0.001** | **1.46** | **[1.26, 1.70]** | **<0.001** |
| Number of obs. | 311 | | | 311 | | |
| R^2^ Marginal/ Conditional | 0.169 / 0.351 | | | 0.269 / 0.502 | | |
| ICC | 0.22 | | | 0.32 | | |

*Note.* Values reflect centered regression coefficients or odds ratios, 95% confidence intervals and *p*-values. ICC, Intra-Class-Correlation. Statistical tests: Linear mixed-effects models with a random intercept for participants.

## **Supplementary Table S3**

*Associations between CUS and episode characteristics, craving, and compliance*

|  | **Predictor** | | |
| --- | --- | --- | --- |
|  | CUS z-score | | |
|  | *Est.* | *CI* | *P_(FDR)_* |
| Total number of entries | **0.57** | **[0.31, 0.83]** | **<0.001** |
| *Ratios of episode types* |  |  |  |
| Type 1 | 0.33 | [0.04, 0.63] | 0.114 |
| Type 2 | 0.07 | [-0.24, 0.38] | 0.875 |
| Type 3 | -0.12 | [-0.44, 0.19] | 0.860 |
| Type 4 | 0.00 | [-0.32, 0.31] | 0.953 |
| Memory vividness | 0.03 | [-0.90, 0.96] | 0.948 |
| Memory intrusiveness | -0.04 | [-0.81, 0.72] | 0.916 |
| Distress | -0.12 | [-0.86, 0.62] | 0.745 |
| Fear | -0.05 | [-0.83, 0.72] | 0.894 |
| Loss of control | 0.37 | [-0.47, 1.22] | 0.386 |
| Craving intensity | -0.69 | [-1.47, 0.09] | 0.083 |
| Compliance rate | -0.18 | [-0.49, 0.13] | 0.257 |
| Discrepancy | 0.29 | [-0.01, 0.59] | 0.057 |

*Note.* Values reflect regression coefficients, 95% confidence intervals and *p*-values. *P*-values for outcomes ratios of episode types were FDR-corrected. Statistical tests: Linear regression models or linear mixed-effects models with a random intercept for participants. CUS, cocaine use severity.

## **Supplementary Table S4**

*Associations between individual components of CUS and episode characteristics, craving, and compliance*

|  | **Predictors** | | | | | | | | |
| --- | --- | --- | --- | --- | --- | --- | --- | --- | --- |
|  | Cocaine use (grams/week; log) | | | Duration of use (years) | | | OCCUS total score | | |
|  | *Est.* | *CI* | *P_(FDR)_* | *Est.* | *CI* | *P_(FDR)_* | *Est.* | *CI* | *P_(FDR)_* |
| Total number of entries | 0.13 | [-0.13, 0.39] | 0.308 | 0.15 | [-0.11 – 0.41] | 0.254 | **0.55** | **[0.29 – 0.81]** | **<0.001** |
| *Ratios of episode types* |  |  |  |  |  |  |  |  |  |
| Type 1 | -0.06 | [-0.36, 0.25] | 0.828 | 0.37 | [0.07, 0.68] | 0.052 | 0.17 | [-0.13, 0.47] | 0.410 |
| Type 2 | 0.23 | [-0.09, 0.56] | 0.317 | 0.05 | [-0.28, 0.37] | 0.775 | -0.18 | [-0.51, 0.14] | 0.410 |
| Type 3 | -0.03 | [-0.36, 0.29] | 0.828 | -0.31 | [-0.62, 0.01] | 0.079 | 0.16 | [-0.16, 0.48] | 0.410 |
| Type 4 | 0.23 | [-0.08, 0.55] | 0.317 | -0.35 | [-0.67, -0.04] | 0.052 | 0.10 | [-0.21, 0.41] | 0.514 |
| Memory vividness | -0.59 | [-1.42, 0.24] | 0.165 | -0.03 | [-0.12, 0.06] | 0.504 | 0.05 | [-0.02, -0.13] | 0.178 |
| Memory intrusiveness | -0.13 | [-0.87, 0.61] | 0.722 | -0.07 | [-0.14, 0.01] | 0.071 | 0.03 | [-0.03, 0.10] | 0.291 |
| Distress | 0.06 | [-0.76, 0.87] | 0.893 | -0.08 | [-0.17, 0.00] | 0.062 | 0.05 | [-0.02, 0.12] | 0.172 |
| Fear | 0.48 | [-0.39, 1.35] | 0.280 | -0.03 | [-0.13, 0.07] | 0.544 | -0.03 | [-0.11, 0.05] | 0.489 |
| Loss of control | 0.06 | [-0.81, 0.92] | 0.894 | -0.06 | [-0.16, 0.03] | 0.163 | 0.03 | [-0.05, 0.11] | 0.471 |
| Craving intensity | -0.05 | [-0.90, 0.81] | 0.913 | **-0.16** | **[-0.25, -0.08]** | **<0.001** | 0.05 | [-0.02, -0.13] | 0.143 |
| Compliance rate | -0.13 | [-0.47, 0.20] | 0.415 | 0.04 | [-0.29, 0.37] | 0.799 | -0.16 | [-0.49, 0.17] | 0.324 |
| Discrepancy | 0.27 | [-0.03, 0.58] | 0.077 | -0.16 | [-0.47, 0.14] | 0.280 | **0.31** | **[0.01, 0.61]** | **0.044** |

*Note.* Values reflect regression coefficients, 95% confidence intervals and *p*-values. *P*-values for ratios of episode types were FDR-corrected within each predictor. Statistical tests: Linear regression models or linear mixed-effects models with a random intercept for participants. CUS, cocaine use severity; OCCUS, Obsessive Compulsive Cocaine Use Scale.

## **Supplementary Table S5**

*Associations between CUS and psychotherapy, and compliance rate and discrepancy*

|  | **Predictors** | | | | | |
| --- | --- | --- | --- | --- | --- | --- |
|  | CUS z-score | | | Psychotherapy experience (no/yes) | | |
|  | *Est.* | *CI* | *P* | *Est.* | *CI* | *P* |
| Compliance rate | -0.11 | [-0.43, 0.20] | 0.473 | -0.25 | [-0.57, 0.06] | 0.110 |
| Discrepancy | 0.28 | [-0.04, 0.59] | 0.084 | 0.06 | [-0.25, 0.38] | 0.679 |

*Note.* Values reflect standardized regression coefficients, 95% confidence intervals and *p*-values. Statistical tests: Linear regression models. CUS, cocaine use severity.

# **Supplementary Section 4: Supplementary Figure**

## **Supplementary Figure S1**

*Note.* Mean ratios of different trigger types for different episode types, averaged across participants. Error bars represent 95% CIs
